# Improved adaptive regularization for simulated annealing inversion of transient electromagnetic

**DOI:** 10.1038/s41598-024-55710-5

**Published:** 2024-03-04

**Authors:** Xiang Tang, Shangbin Liu, Xiaofei Nian, Shengqiang Deng, Yuchao Liu, Qiongyao Ye, Yingjie Li, Yangyi Li, Tong Yuan, Huaifeng Sun

**Affiliations:** 1 Guangxi Communications Design Group Co., Ltd., Nanning, China; 2 Geotechnical Engineering Research Center, Shandong University, Jinan, China; 3 Laboratory of Earth Electromagnetic Exploration, Shandong University, Jinan, China

**Keywords:** Transient electromagnetic, Simulated annealing, Adaptive regularization, Inversion, Solid Earth sciences, Geophysics

## Abstract

Geophysical inversion usually involves ill-posed problem. Regularization is the most commonly used method to mitigate this problem. There are many regularization parameter selection methods, among which the adaptive regularization method can automatically update parameters during iteration, reducing the difficulty of parameter selection. Therefore, it is widely used in linear inversion. However, there are very few studies on the use of adaptive regularization methods in stochastic optimization algorithms. The biggest difficulty is that in stochastic optimization algorithms, the search direction of any iteration is completely random. Data fitting term and stabilizing term vary in a wide range, making it difficult for traditional methods to work. In this paper, we consider the contributions of the data fitting term and the stabilizing term in the objective function and give an improved adaptive regularization method for very fast simulated annealing (VFSA) inversion for transient electromagnetic (TEM) data. The optimized method adjusts the two terms dynamically to make them in balance. We have designed several numerical experiments, and the experimental results demonstrate that the method in this paper not only accelerates the convergence, but also the inversion results are very little affected by the initial regularization parameter. Finally, we apply this method to field data, and the inversion results show very good agreements with nearby borehole data.

## Introduction

Inversion plays a vital role in the interpretation of electromagnetic data. Conventional descent-based inversion methods search the direction toward a smaller fitting error. The success of these methods is heavily dependent on the quality of the initial model. If the initial model is appropriate, these methods can converge to the global optimum quickly; otherwise, they may converge to a local minimum^1,2^. To avoid this problem, various global optimization methods independent of the initial models have been proposed, such as the simulated annealing (SA) algorithm^3^; Genetic algorithm (GA)^4^. The simulated annealing algorithm simulates the process of liquid cooling crystallization. When the temperature is high, the molecules can move freely, and the whole system is in a chaotic state. As the temperature decreases, the system gradually becomes more orderly. At the lowest temperature, the molecules are no longer able to move, and the energy of the system is at its lowest level. During the iterative process, the SA algorithm is designed to not only receive better solutions but also receive poor solutions with a certain probability. The proposed approach enhances the search capability and enables the algorithm to escape local minima, thereby facilitating the discovery of global minima. However, the convergence of SA is very slow. The very fast SA (VFSA) is an improvement of SA^5^. It replaces Gibbs distribution with Cauchy distribution and uses an exponential cooling strategy. These improvements greatly improve the convergence speed of VFSA algorithm. So, VFSA has been widely used in geophysical field^6–9^.

Before inversion, we must to determine suitable parameters. Specific to transient electromagnetic (TEM) problem, we need to evaluate layer number and resistivity range. These works are usually done through priori information or rapid imaging. Due to technical limitations or complex geology, the selected parameters are not always appropriate. This will inevitably cause the inversion results to deviate significantly from the true model. Our approach in this paper will use a sufficient number of parameters for SA, rather than relying on manual selection. However, the increase of parameters will affect the inversion quality, increase the time cost, and worsen the nonuniqueness problem of the inversion^10^. Tikhonov regularization methods are widely used in geophysical inversions to mitigate the multiplicity and help algorithm convergence^11–13^.

The regularization parameter balances the data fitting and the stabilizing terms, which ultimately affects the inversion results. How to choose the appropriate regularization parameter is a crucial aspect in inversion. There exist various techniques for selecting the regularization parameter, such as Generalized cross-validation method^14^, and the L-curve method^15^. Although these methods are effective, they are also time-consuming. After that, some easier adaptive regularization methods have been developed. Zhdanov (2002) presents an adaptive method to make the regularization parameters decay exponentially according to an empirical attenuation coefficient. Chen et al^16^. proposed an adaptive regularization method, which used the ratio of the data fitting term to the sum of the data fitting term and the stabilizing term as the regularization parameter in subsequent iterations. The implementation of adaptive regularization techniques in linear inversion problems has been widely studied^17–19^. However, there have been few discussions regarding the utilization of these techniques in stochastic optimization algorithms. Chen et al^20^. used the SA algorithm with regularization method to synchronous interpretation of seismic and magnetic data. However, their research lacked research on the effect of regularization parameter selection on inversion efficiency and stability, which is crucial for stochastic optimization algorithms. In VFSA, the perturbation range of the model decreases as the temperature decreases^21^. It means that if the regularization parameter is inappropriate, VFSA algorithm will require more steps and more time to get a better model. A new method that produces an adaptive regularization parameter with better stability and higher computational efficiency is needed.

In this paper, we propose an improved adaptive regularization method for VFSA. The ratio of the data fitting term to the stabilizing term is used as a criterion to assess the appropriateness of the current regularization parameter. We keep the balance by adjusting the regularization parameter, preventing model bias towards one item. The great advantage of this method is that with the help of the regularization term, the convergence efficiency is substantially improved due to the narrowing of the model space while the total objective function converges. To test the effectiveness of this improvement, we apply it to a synthetic three-layered geological model and compare the inversion results with the original SA algorithm. The experimental results show that our method is more stable and less affected by noise compared to the original SA algorithm. Then, we designed three sets of experiments using the same model and different initial regularization parameters to test the adaptive capability of this method. Experimental results show that our method is little affected by the initial regularization parameter, and the convergence speed is dramatically improved. We have also tested other synthetic models, and the results all show that the inverse models of this method agree well with the true models. Finally, we apply the method to field data. The results show good agreement between the inversion model and nearby borehole data.

## Regularization methodology

According to Tikhonov regularization theory^22,23^, the objective function can be written as a linear combination of the data fitting term and the stability term as follows,

1 $$ P\left( {\text{m}} \right){ = }\Phi \left( {\text{m}} \right) + \lambda S\left( {\text{m}} \right) = \left\| {d - A{\text{m}}} \right\|^{2} + \lambda \left\| {H\left( {{\text{m}} - {\hat{\text{m}}}} \right)} \right\|^{2} $$

where P is the the objective function, Φ and S are data fitting term and the stability term , respectively. **m** is the model parameter vector, **λ** is the regularization parameter, **d** and A are the observed data and forward operators, respectively. H is the smoothness matrix, and $\widehat{{\text{m}}}$ is a reference model. In the absence of a reference model, $\widehat{{\text{m}}}=0$.

The regularization parameter plays an important role in balancing the data fitting term and the stabilizing term. When λ → 0, the data fitting term plays a major role in the objective function. When λ → ∞, the stabilizing term is dominant, and the inversion process will be mainly driven by prior information. In literatures^17,24^, the initial regularization parameter is the ratio of the data fitting term to the stabilizing term of the initial model, and the attenuation coefficient is selected by experience. This method is effective in linear inversion methods, but not in SA algorithm due to its random search direction. As the temperature decreases in VFSA, the perturbation range of the model shrinks. If the regularization parameters at lower temperatures are not appropriate, the model may be biased towards either the data fitting term or the stabilizing term. Then, in subsequent iterations, SA will need more time and steps to jump out of the current solution to find a better one. In more severe cases, the SA algorithm may not be able to converge to the global optimal within a limited time.

In this study, we define a criterion to evaluate the deviation of the current solution from a reasonable range to the data fitting term or the stabilizing term, and dynamically adjust the regularization parameter according to the deviation degree of the current solution. This balances the two terms in the objective function during the cooling process. The regularization parameter updates its value as follows,

2 $$ \lambda^{k + 1} = \left\{ {\begin{array}{*{20}c} {\lambda^{k} } & {0 < \frac{{{\Phi }\left( {m_{k} } \right)}}{{{\text{S}}\left( {m_{k} } \right)}} \le \varphi_{1} } \\ {a_{1} \lambda^{k} } & {\varphi_{{1}} < \frac{{{\Phi }\left( {m_{k} } \right)}}{{{\text{S}}\left( {m_{k} } \right)}} \le \varphi_{2} } \\ {a_{2} \gamma^{k} } & {\varphi_{2} < \frac{{{\Phi }\left( {m_{k} } \right)}}{{{\text{S}}\left( {m_{k} } \right)}} } \\ \end{array} } \right. $$

where *k* denotes the *k*_*th*_ iteration, $m_{k}$ is the current best model. $\varphi_{1}$ and $\varphi_{2}$ are used to judge the degree of the deviation of the current solution from a reasonable range, and can take some empirical values by a large number of numerical simulations. $\varphi_{1} \in \left[ {0.1,0.2} \right]$ and $\varphi_{2} \in \left[ {0.9,1.0} \right]$ in this study to keep $\Phi \left( {m_{k} } \right)$ and $S\left( {m_{k} } \right)$ with the same order of magnitude. $a_{1}$ and $a_{2}$ are empirical attenuation coefficients, where $a_{1}$ and $\alpha_{2} \in \left[ {0.5,0.9} \right]$ and ${ }a_{1} > a_{2}$. In the following computation, $a_{1} = 0.8$ and $a_{2} = 0.6$. The initial model is generated randomly.

Generally, we use a large value as the initial regularization parameter, and then automatically update the regularization parameter in the next iteration. In Eq. (2), when the value of ${{\Phi \left( {m_{k} } \right)} \mathord{\left/ {\vphantom {{\Phi \left( {m_{k} } \right)} {S\left( {m_{k} } \right)}}} \right. \kern-0pt} {S\left( {m_{k} } \right)}}$ is between $\varphi_{1}$ and $\varphi_{2}$, we believe that the data fitting term and the stabilizing term are balanced, and $\lambda$ is appropriate for the current iteration. We slow down the decrease of $\lambda$ in the next iteration by letting $\lambda^{k + 1} = a_{1} \lambda^{k}$. When the value of ${{\Phi \left( {m_{k} } \right)} \mathord{\left/ {\vphantom {{\Phi \left( {m_{k} } \right)} {S\left( {m_{k} } \right)}}} \right. \kern-0pt} {S\left( {m_{k} } \right)}}$ is between 0 and $\varphi_{1}$, the data fitting term decreases faster than the stabilizing one in the previous iterations. It seems that the previous regularization parameters were not effective due to their small value. Therefore, we freeze the regularization parameter in the next iteration($\lambda^{k + 1} = \lambda^{k}$). On the other hand, when the value of ${{\Phi \left( {m_{k} } \right)} \mathord{\left/ {\vphantom {{\Phi \left( {m_{k} } \right)} {S\left( {m_{k} } \right)}}} \right. \kern-0pt} {S\left( {m_{k} } \right)}}$ is greater than $\varphi_{2}$, the data fitting term decreases slower than the stabilizing one in the previous iterations. This means that the value of the previous regularization parameters are too large. Therefore, we decrease the regularization parameter faster in the next iteration by letting $\lambda^{k + 1} = a_{2} \lambda^{k}$.

Due to the models generated in VFSA are random, the ratio ${{\Phi \left( {m_{k} } \right)} \mathord{\left/ {\vphantom {{\Phi \left( {m_{k} } \right)} {S\left( {m_{k} } \right)}}} \right. \kern-0pt} {S\left( {m_{k} } \right)}}$ is in a quite large dynamic range. This may cause the current λ to become completely invalid. To circumvent such problem, we add the following restriction

3 $$ \frac{{\Phi \left( {m_{k} } \right)}}{{P\left( {m_{k} } \right)}} < \beta $$

where $\beta$ is set to be 0.9 empirically. Equation (3) is used to prevent the stabilizing term from failing due to a very small regularization parameter. In the inversion process, we first ensure that the stability term is still valid through Eq. (3). Then, we use Eq. (2) to update the regularization parameter. Vice versa, if Eq. (3) is not satisfied, it means the stabilizing term fails. It is necessary to freeze the regularization parameter. There are several coefficients involved in the above process, but they do not need to be precise. Once the form of the objective function is determined, these parameters are appropriate as long as they are in a certain interval.

### Synthetic examples

To test the effectiveness of the method proposed in this paper, a three-layered model with a low resistivity layer in the middle (H model), is used. The detailed model parameters are listed in Table 1. The transmitter is a 100 m × 100 m loop with 1 Ampere current. The initial regularization parameter value λ_0_ is 2. In this inversion, we use a random model with 10 layers of equal thickness as the starting model. For the convenience of comparison, we keep the thickness of the layer unchanged throughout the inversion process in this experiment and only invert the layer conductivity. In later studies, we invert resistivity and layer thickness together.

**Table 1:** Used parameters of three-layered and five-layered models.

Model #	ρ (ohm-m)					h (m)			
	ρ_1_	ρ_2_	ρ_3_	ρ_4_	ρ_5_	h_1_	h_2_	h_3_	h_4_
H	200	50	150	–	–	200	70	–	–
K	100	300	100	–	–	200	100	–	–
HKH	300	50	300	50	300	100	100	100	100
KQH	100	300	100	30	100	100	100	100	100

The layer thickness is 37 m. The resistivity for each layer is in the range [20, 400]. There are 10 parameters (layer resistivity) to be inverted. We add 5% Gaussian noise to the data, following a typical noise model in TEM proposed by Munkholm and Auken (1996) and the error floor suggested in the literature^25^. The noise model is shown below:

4 $$ {{{\text{d}}Bz} \mathord{\left/ {\vphantom {{{\text{d}}Bz} {dt = bt^{a} }}} \right. \kern-0pt} {dt = bt^{a} }} $$

where b is a proportional coefficient and is a constant. t is the time after the current is turned off, and a is the slope. In this paper, $a = - {1 \mathord{\left/ {\vphantom {1 2}} \right. \kern-0pt} 2}$.

**Figure 1:**
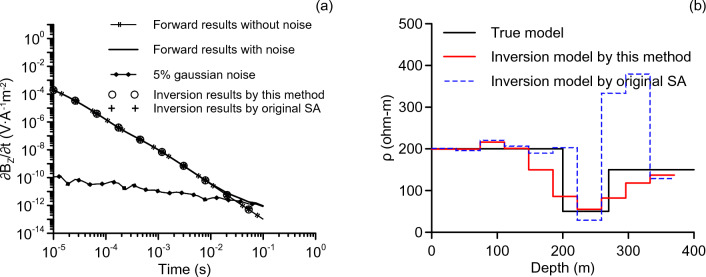
Inversion results of original SA and the method proposed in this paper. The attenuation voltage curves are shown in (**a**). The inversion models are shown in (**b**).

To ensure comparison validity, we run each inversion 10 times. Each run includes 100 iterations and 20 random steps per iteration. Then, we calculate the mean value of the 10 individual results as the final result. The final result is presented in Fig. 1. Figure 1a displays the attenuation voltage curve, while Fig. 1b represents the inversion model. It can be seen from Fig. 1a that the inversion data of this improved method and that of the original SA method (without regularization) are in good agreement with the forward data. However, the inversion models (present in Fig. 1b) show significant differences. The inversion model of the improved method is closer to the true model, whereas the inversion model of the original SA shows a false structure in the deep, deviating from the true model. This experiment proves that the inversion has multiple solutions. Moreover, the inclusion of regularization terms can help the algorithm to converge to the true model, even if the data is contaminated by noise.

**Figure 2:**
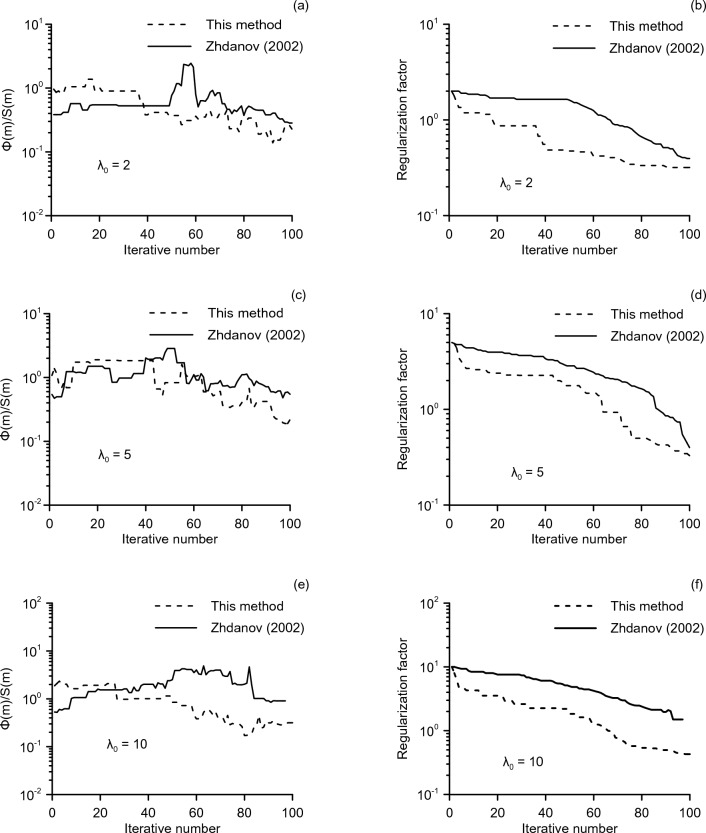
Φ(m_k_)/S(m_k_) (left column) and regularization factor (right column) with iterations from different initial regularization parameters. Three rows are using initial regularization value 2, 5, and 10 respectively.

To further test the performance of the improved method, we compared the inversion parameters in SA algorithm with different regularization methods. In this experiment, the influence of different initial regularization factors: 2, 5 and 10 on the inversion process is studied, and the adaptability of the regularization method for different initial values is tested. In the traditional method, the regularization factor is calculated as a cooling process. When the RMS decreases rapidly, the regularization parameter will hold for the next iteration. When the RMS decreases slowly, it will decay in the next iteration by multiplying by an factor less than one. However, in the SA algorithm, due to its stochastic nature, the RMS at the end of each iteration may not only decrease but also grow. This makes it impossible to update the regularization factor according to the traditional method, which is the purpose of this paper to propose an improved method for updating the regularization factor. In order to test the effectiveness of the proposed method, the regularization factors in the comparison experiments are no longer updated with reference to the RMS, but are simply updated based on iterations. The results of the experiment are displayed in Fig. 2. The left column shows the ratio ${{\Phi \left( m \right)} \mathord{\left/ {\vphantom {{\Phi \left( m \right)} {S\left( m \right)}}} \right. \kern-0pt} {S\left( m \right)}}$ obtained by the regularization method in this paper and in Zhdanov (2002). The right column shows how the regularization factors change with iteration.

From the three graphs in the left column in Fig. 2, we can see that during the iterative process, the ratio ${{\Phi \left( m \right)} \mathord{\left/ {\vphantom {{\Phi \left( m \right)} {S\left( m \right)}}} \right. \kern-0pt} {S\left( m \right)}}$ of the improved method slowly decays until it converges. In contrast, when the initial regularization factor is large (e.g., $\lambda_{0} = 10$), the ratio ${{\Phi \left( m \right)} \mathord{\left/ {\vphantom {{\Phi \left( m \right)} {S\left( m \right)}}} \right. \kern-0pt} {S\left( m \right)}}$ of the traditional method is larger in the late period than in the early period. The right column of Fig. 2 shows the regularization factor changes with iteration. It can be observed that the regularization factors in the improved method are almost the same in the later iterations, despite from different initial values. In contrast, the regularization factors in Zhdanov (2002) exhibit significant differences in the later iterations when the initial values are different.

**Figure 3:**
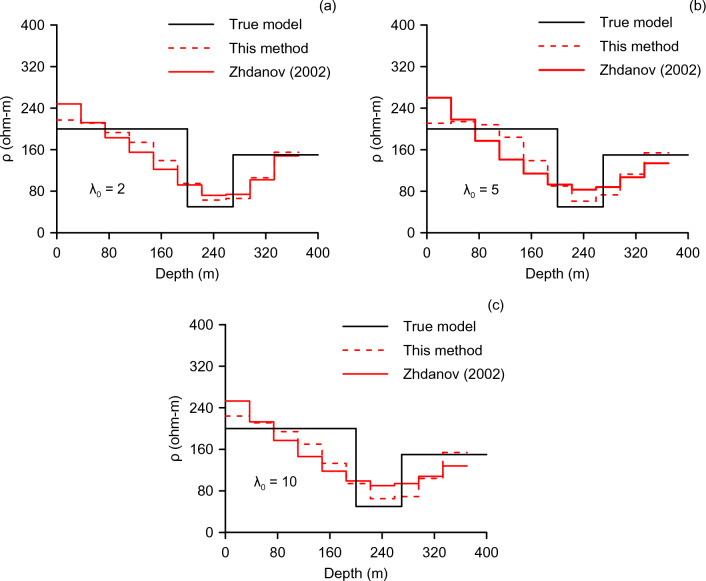
Inversion result using different regularization methods. (**a**), (**b**), and (**c**) are the results from initial regularization parameter 2, 5, and 10, respectively.

Figure 3 shows the inversion results using different regularization methods. The black lines represent the true model. The red solid line and the red dashed line are the inversion results using different regularization methods. Figure 3a, b and c correspond to the different initial value λ_0_ = 2, 5, 10, respectively. As can be seen in Fig. 3, the inversion results are all in good agreement with the true model. And the results of the improved regularization method has a better resistivity fit for the top layer and the bottom background, and it has a lower resistivity value in the middle layer, which is closer to the true model. Moreover, the improved method gives almost the same models, although the initial regularization values are different. On the other hand, the results obtained using conventional regularization methods are subtly different. The larger the initial regularization parameters used, the smoother the inversion results. The root mean square (RMS) results with different strategies are shown in Table 2. As can be seen, all the results of this paper’s method are better than the traditional method. It is worth noting that this experiment illustrates that the inversion results of the improved method proposed in this paper are almost unaffected by the initial regularization parameter, while the inversion results of the traditional method are strongly influenced by the initial regularization parameter. This facilitates our choice of initial parameter. Because, there is a wider range to choose from, larger or smaller ones are all suitable.

**Table 2:** RMS results.

Methods	RMS (λ_0_ = 2)	RMS (λ_0_ = 5)	RMS (λ_0_ = 10)
This method	0.00346	0.00561	0.00457
Zhdanov	0.00942	0.01434	0.01955

**Figure 4:**
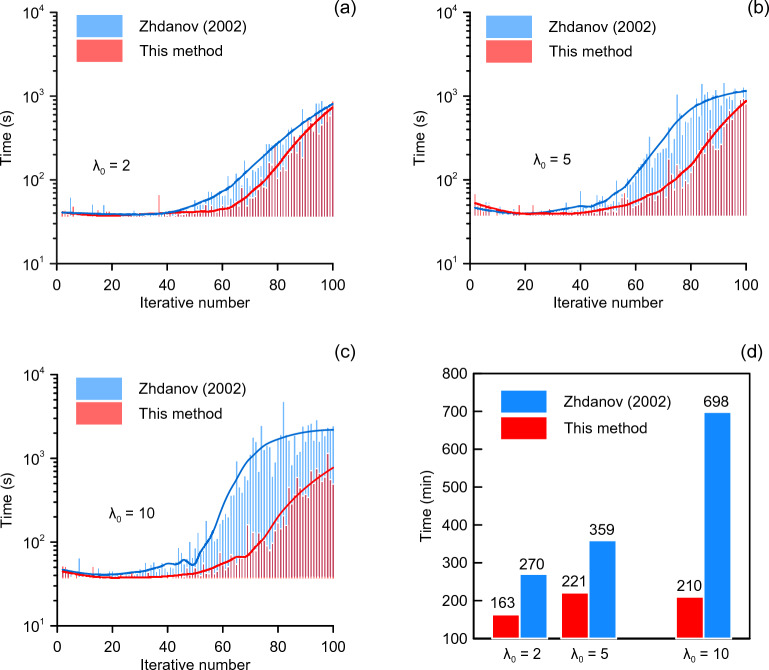
Time cost between the improved and the conventional regularization method. (**a**), (**b**), and (**c**) are time costs in each step using different regularization methods. The blue and red curves in the figures are the fitting curve, and these curves correspond to the time cost trend. (**d**) shows the total time cost of inversion using different regularization methods.

Although all inversion results are acceptable, the computational efficiency can vary significantly. Figure 4 presents the time costs in each iteration step using different regularization methods when the initial values are different. We have plotted the trend curves for time costs using both methods in Fig. 4a, b and c, with blue and red lines. earlier iterations, the two lines are almost identical without any noticeable difference. However, in later iterations, the two lines diverged significantly from each other. After more than 20 iterations, the time of each iteration used by the method in this paper is obviously less than that of the other method. Figure 4d shows the total time cost using two different methods. For three different initial regularization values, the total time cost of the proposed method is significantly faster than that of the traditional method by 1.6, 1.6 and 3.3 times. The convergence time of the improved regularization method has a weak relationship with λ_0_. Our FORTRAN code runs on a PC with Intel Core i5-7400 CPU (3.0 GHz) and 8 GB RAM.

**Figure 5:**
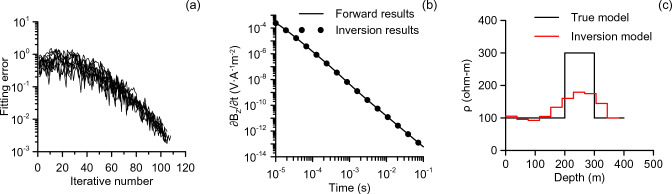
Inversion results for the K model. (**a**) The data fitting error of 10 runs; (**b**) attenuation voltage curve; (**c**) inversion model.

We apply this method to invert a K type model with a high resistivity layer in the middle. The detailed model parameters are listed in Table 1. The transmitter is still a 100 m × 100 m loop with 1 Ampere current. The resistivity for each layer is in the range [20, 400]. The layer thickness for each layer is in the range [30, 40]. The number of parameters to be inverted this time increases to 11: 10 layer conductivity and one layer thickness. we run each inversion 10 times, with 110 iterations for each run and 20 random steps for each iteration. The mean value of 10 runs is used as the final inversion results. Figure 5 shows the inversion results and the inversion parameter during the iterative process. Figure 5a shows how the data fitting error changes with the number of iterations during 10 runs. In the early iterations, the results of each run varied significantly and fluctuated over a wide range. With the late iteration, the data fitting error of each run changes little, and the algorithm gradually converges. Figure 5b illustrates the induced potential observed at the center of the loop. It can be observed that the inversion results are in good agreement with the forward data. The inversion model is illustrated in Fig. 5c, from which it can be seen that all the parameters of the model are well recovered except for the conductivity of the middle high resistance layer. The high resistance layer does not recover well, which can be attributed to the characteristics of the electromagnetic method, which is more sensitive to low resistance than high resistance. Generally, the inversion model can reflect the characteristics of the true model.

**Figure 6:**
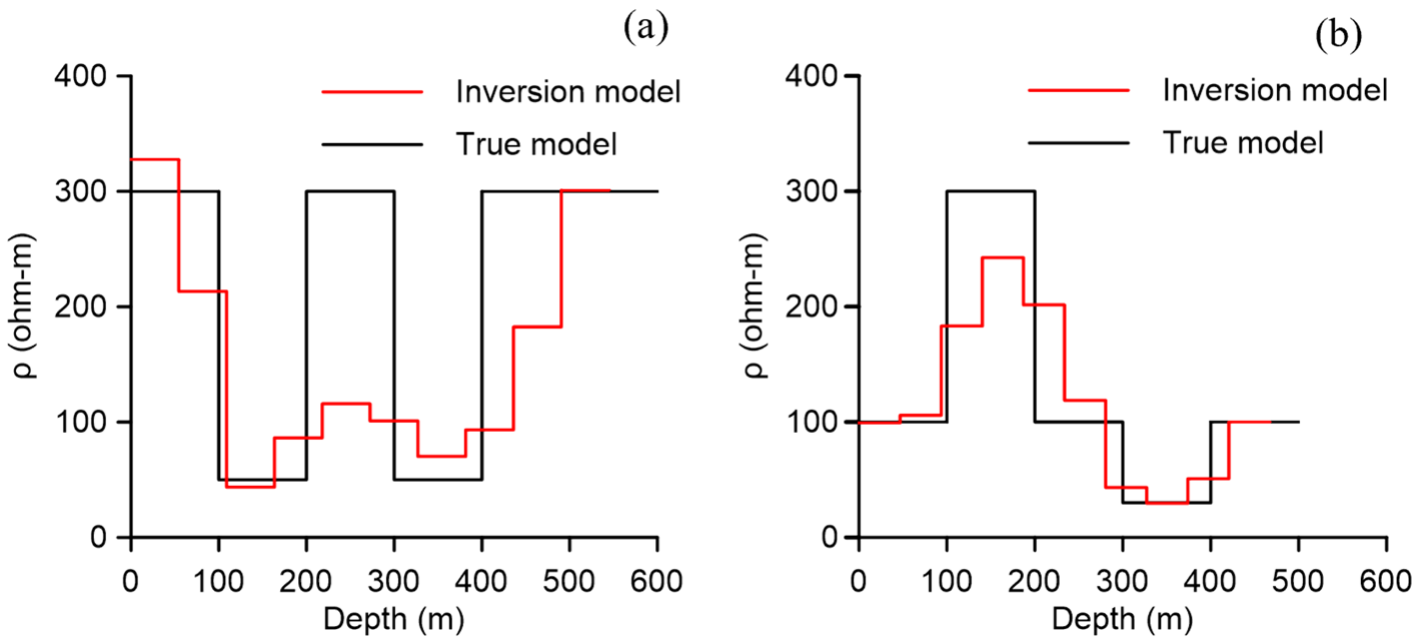
Inversion results for complex five-layered models, (**a**) HKH and (**b**) KQH.

We apply the improved method to invert two 5-layered models: a HKH model and a KQH model. The detailed model parameters are listed in Table 1. A 300 m × 300 m rectangular loop is used as a transmitting source with the current of 1A. A 10-layer model with the same layer thickness is used, and we inverted the layer thicknesses and the conductivity of each layer. The resistivity to be inverted is in the range [20,400], and the layer thickness to be inverted is in the range [45,55]. we run each inversion 10 times, with 110 iterations for each run. The number of random steps for each iteration increases to 40. The mean value of 10 runs is used as the final inversion result and shown in Fig. 6. The inversion models all better reflect the characteristics of the true models, from which the low and high resistance layers can be well distinguished. However, in the K-like part of the complex model (two low resistance layers sandwiched by a high resistance layer), the resistivity of the middle layer does not fit well. The specific reasons have already been discussed above.

## Case history

**Figure 7:**
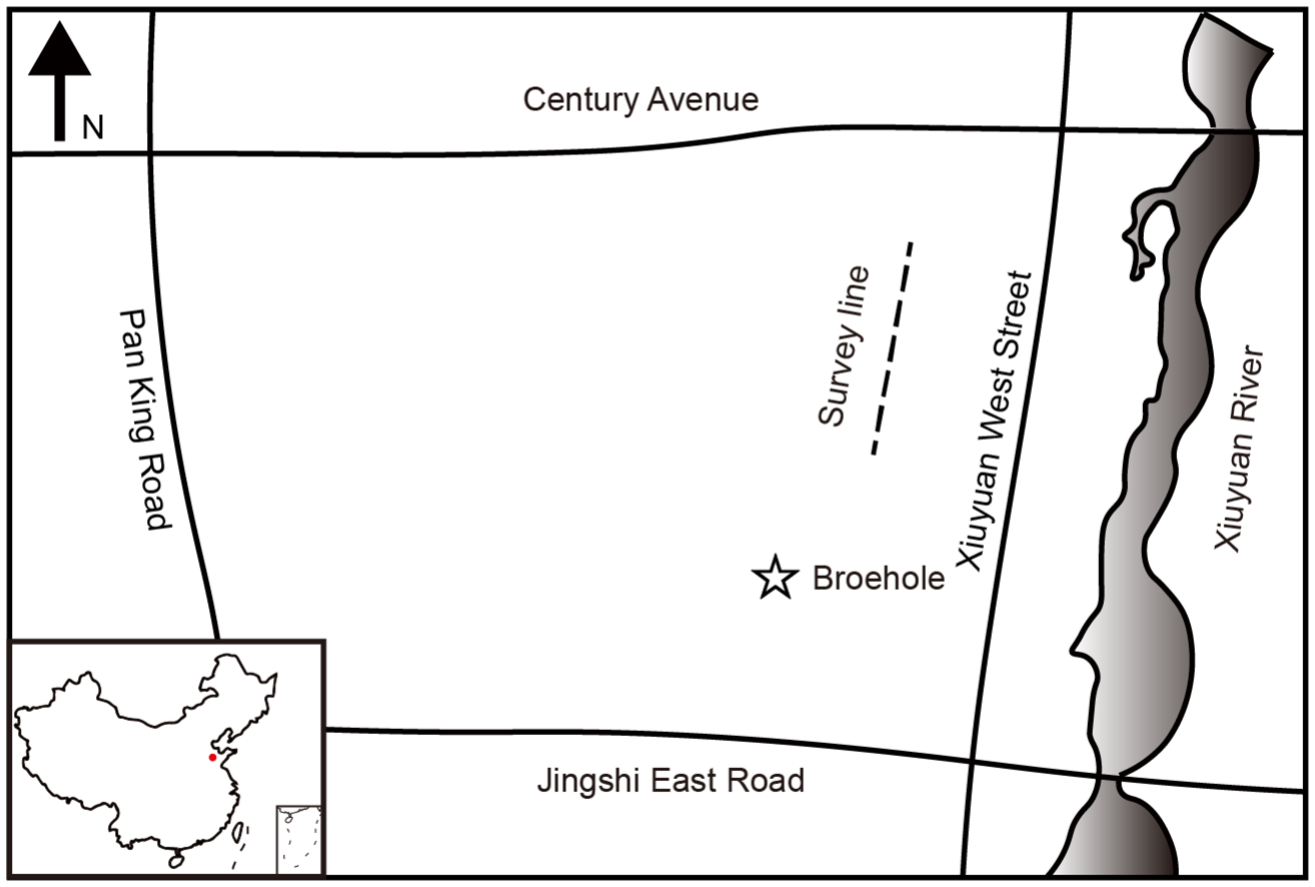
Schematic diagram of the field site position. The position of the survey line is marked with dashed line in the west of the river. The star indicates the location of a known borehole.

To further test the method presented in this paper, we invert TEM field data. The data is acquired in Jinan, Shandong Province, China. The survey line is located to the west of Xiuyuan River, between Jingshi East Road and Century Avenue, as shown by the dotted line in Fig. 7. There are 24 stations in the profile with a spacing of 25 m. A existing borehole with a depth of 130 m is very close to the survey line, marked as a star in Fig. 7. We use this borehole data to verify our inversion results in the near-surface portion. We use a 100 × 100 m loop as the transmitter. The measured data are received at the center of the transmitting loop and are normalized with the transmitting current and the receiver coil area. We use a random 10-layer initial model with the same layer thickness and invert the layer resistivity and thicknesses.

**Figure 8:**
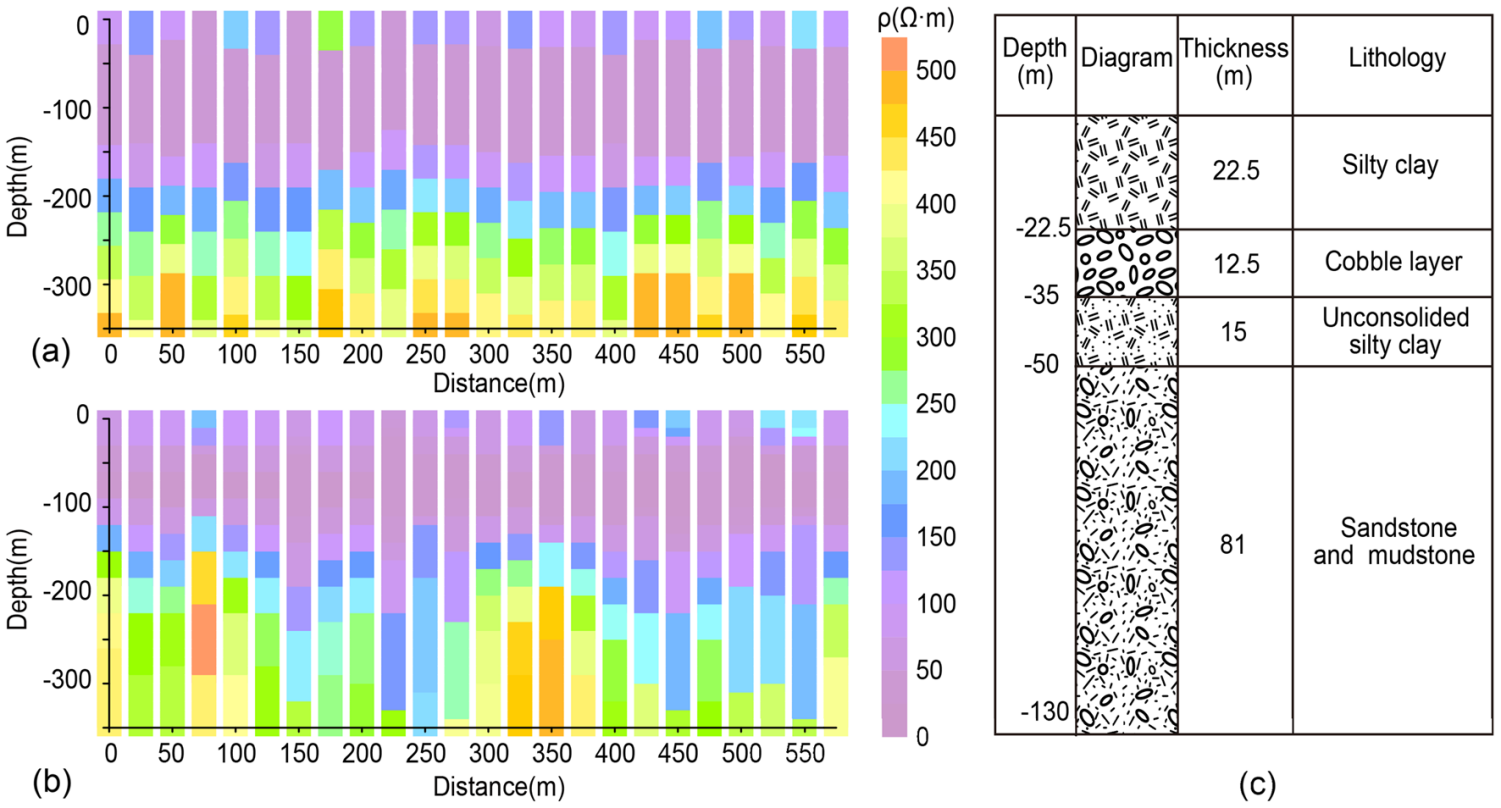
(**a**) Resistivity profile inverted by the method presented in this paper, (**b**) resistivity profile inverted by the 1D smooth method, and (**c**) the nearby borehole records.

We invert the field data for all stations in the profile using the improved method in this paper and the 1D smooth inversion method. Figure 8a, b displays the resistivity profile of the inversion while Fig. 8c illustrates the borehole data, providing information from the surface up to 130 m depth. The stratigraphic distribution revealed by the borehole data can be roughly divided into four layers. From the surface to a depth of − 22.5 m, the earth is composed of consolidated silty clay. Between depths of − 22.5 m to − 35 m, there is cobble. At a depth of − 35 m to − 50 m, there is a layer of silty clay that is not fully consolidated. Below this layer are strata of sandstone and mudstone with poor cementation and fracture development. Based on the borehole data and nearby well investigation, the water table is approximately 10–30 m below the ground surface. Therefore, non-dense stratums below 30 m will be filled with water and show low resistivity in TEM results.

**Figure 9:**
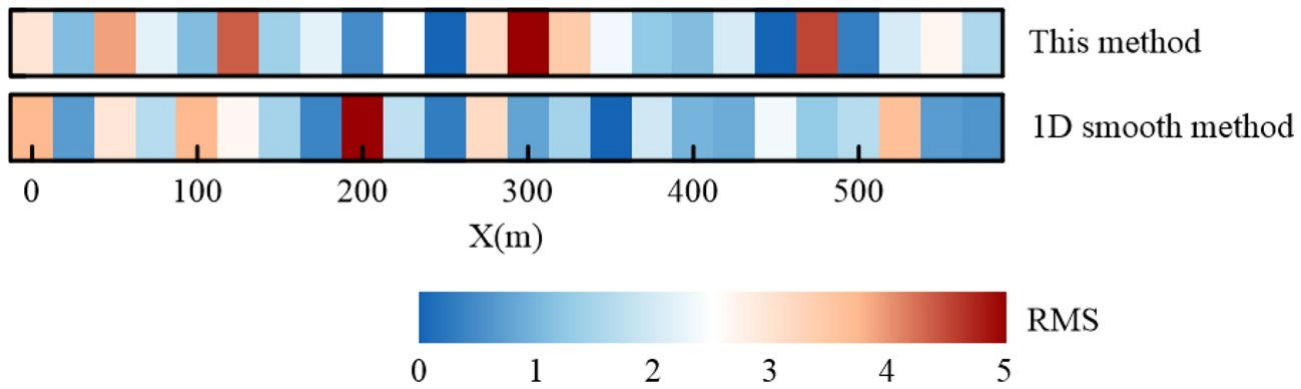
RMS.

We can see that the stratum can be roughly divided into three layers from the two inversion profiles. The first layer presents high resistance characteristics and is about 40 m thick. The second layer presents a very obvious feature of low resistance, the resistivity is less than 130 Ω m, and the depth is approximately − 40 ~  − 160 m. There is a high resistance layer below − 160 m. The inversion results of the two methods are consistent with the local geological conditions revealed by borehole data. However, When the fitting error of the two methods is essentially consistent, we can find that result of the improved method is clearer than that of the 1D smooth inversion method for the interface between the first and second layers. Additionally, compared to the results of the 1D smooth inversion method, the results of this paper’s method have a more continuous resistivity distribution between measurement points. These match the real geology a little better. Figure 9 shows the RMS of each measurement stations, and it can be seen that the majority of the results of both methods are below 5%.

## Conclusions

We present an improved adaptive regularization method for VFSA. This method solves the issue of traditional regularization method failures caused by random search direction of complete nonlinear inversion. In this method, the ratio of data fitting term and stable term is used as the criterion to judge whether the current regularization factor is suitable, and the regularization factor is updated according to the judgment result. Different models are used to test the improved method, and the results show that the inversion results of the improved method are better than the traditional method, and the calculation efficiency is high. The improved method is not sensitive to the initial regularization values and has good adjustment ability. Finally, we test the algorithm with a TEM field data. The inversion results are in good agreement with the real stratum distribution revealed from a nearby borehole, and the results can distinguish the stratum more easily than the results of the 1D smooth inversion method. This method can also be used in other stochastic optimization algorithms.

## Data Availability

The datasets generated and analysed during the current study are not publicly available due confidential but are available from the corresponding author on reasonable request.
